# Detection of Influenza Virus Using a SOI-Nanoribbon Chip, Based on an N-Type Field-Effect Transistor

**DOI:** 10.3390/bios11040119

**Published:** 2021-04-12

**Authors:** Kristina A. Malsagova, Tatyana O. Pleshakova, Andrey F. Kozlov, Rafael A. Galiullin, Vladimir P. Popov, Fedor V. Tikhonenko, Alexander V. Glukhov, Vadim S. Ziborov, Ivan D. Shumov, Oleg F. Petrov, Vladimir M. Generalov, Anastasia A. Cheremiskina, Alexander G. Durumanov, Alexander P. Agafonov, Elena V. Gavrilova, Rinat A. Maksyutov, Alexander S. Safatov, Valentin G. Nikitaev, Alexander N. Pronichev, Vladimir A. Konev, Alexander I. Archakov, Yuri D. Ivanov

**Affiliations:** 1 Laboratory of Nanobiotechnology, Institute of Biomedical Chemistry, 119121 Moscow, Russia; t.pleshakova1@gmail.com (T.O.P.); afkozlow@mail.ru (A.F.K.); rafael.anvarovich@gmail.com (R.A.G.); ziborov.vs@yandex.ru (V.S.Z.); shum230988@mail.ru (I.D.S.); alexander.archakov@ibmc.msk.ru (A.I.A.); yurii.ivanov.nata@gmail.com (Y.D.I.); 2 Rzhanov Institute of Semiconductor Physics, Siberian Branch of Russian Academy of Sciences, 630090 Novosibirsk, Russia; popov@isp.nsc.ru (V.P.P.); ftikhonenko@gmail.com (F.V.T.); 3 JSC Novosibirsk Plant of Semiconductor Devices with OKB, 630082 Novosibirsk, Russia; gluhov@nzpp.ru; 4 Joint Institute for High Temperatures of Russian Academy of Sciences, 125412 Moscow, Russia; ofpetrov@ihed.ras.ru; 5 Federal Budgetary Research Institution—State Research Center of Virology and Biotechnology VECTOR, Federal Service for Surveillance on Consumer Rights Protection and Human Well-Being, 630559 Novosibirsk Region, Koltsovo, Russia; general@vector.nsc.ru (V.M.G.); cheremiskina_aa@vector.nsc.ru (A.A.C.); motoknerva@gmail.com (A.G.D.); agafonov@vector.nsc.ru (A.P.A.); gavrilova_ev@vector.nsc.ru (E.V.G.); maksyutov_ra@vector.nsc.ru (R.A.M.); safatov@vector.nsc.ru (A.S.S.); 6 National Research Nuclear University MEPhI (Moscow Engineering Physics Institute), 115409 Moscow, Russia; vgnikitayev@mephi.ru (V.G.N.); kaf46@mail.ru (A.N.P.); 7 Department of Infectious Diseases in Children, Faculty of Pediatrics, Pirogov Russian National Research Medical University, 117997 Moscow, Russia; konev60@mail.ru

**Keywords:** SOI, nanoribbon, silicon-on-insulator, influenza A virus, antibody

## Abstract

The detection of influenza A virions with a nanoribbon detector (NR detector) has been demonstrated. Chips for the detector have been fabricated based on silicon-on-insulator nanoribbon structures (SOI nanoribbon chip), using a complementary metal-oxide-semiconductor (CMOS)-compatible technology—by means of gas-phase etching and standard optical photolithography. The surface of the SOI nanoribbon chip contains a matrix of 10 nanoribbon (NR) sensor elements. SOI nanoribbon chips of n-type conductance have been used for this study. For biospecific detection of target particles, antibodies against influenza virus have been covalently immobilized onto NRs. Influenza A virus detection was performed by real-time registration of the source-drain current through the NRs. The detection of the target viral particles was carried out in buffer solutions at the target particles concentration within the range from 10^7^ to 10^3^ viral particles per milliliter (VP/mL). The lowest detectable concentration of the target viral particles was 6 × 10^−16^ M (corresponding to 10^4^ VP/mL). The use of solutions containing ~10^9^ to 10^10^ VP/mL resulted in saturation of the sensor surface with the target virions. In the saturation mode, detection was impossible.

## 1. Introduction

Worldwide, A-group influenza virus is the cause of over 9 million cases of hospitalization and around 6.6 million deaths annually [1,2]. Highly efficient anti-influenza drugs are already being developed and mass-produced. At the same time, early diagnosis at the initial stage of the disease allows one to provide timely medical treatment, avoiding the development of severe acute forms of the disease (which can cause death). Moreover, early diagnosis of influenza infection allows for timely anti-epidemic activities, thus minimizing the social, economic, and political consequences of the disease. This is why the development of highly sensitive methods of influenza virus detection represents a problem of modern medicine.

Influenza viruses pertain to the *Orthomyxoviridae* family, and are divided into several types—A, B, C, and D—based on antigen differences in the virion’s ribonucleoproteins, the latter not starting any inter-type serologic reactions, and defining the virus’ category. Seasonal epidemics are caused by influenza A and influenza B viruses. Virions of these viruses (with a diameter of 80–120 nm, though they are sometimes filamentous and reach a length of over 20 µm) consist of a two-layer envelope and are covered by multiple transmembrane glycoproteins—hemagglutinin (HA) and neuraminidase (NA), as well as by small (20–60 molecules/virion) quantities of M2 protein. M1 membrane protein, being one of the most abundant proteins in a virion, connects to the lipid envelope to maintain virion morphology. A nucleocapsid inside the virion is formed by eight fragments of single-stranded RNA, a nucleoprotein (N), and polymerase complex proteins (PA, PB1, and PB2) [3,4]. The number of copies of each protein can vary for different virus strains [4].

To date, enzyme-linked immunosorbent assay (ELISA)-based, and polymerase chain reaction (PCR)-based methods have become popular in the laboratory diagnosis of influenza [5,6]. However, the analysis time required to obtain results using these methods, is 4 to 6 h, which is too long in the case of urgent anti-epidemic measures.

Nanoribbon (NR) detector represents a molecular detector, which allows one to identify single biological macromolecules and viral particles [7] in the course of their counting, which defines the high speed and concentration sensitivity of analysis. The principle of operation of the NR detector is based on the registration of an electric current flowing through a NR. A viral particle (VP), upon its adsorption onto the NR surface, changes its conductivity. Chiang et al. [8], and Patolsky et al. [7], demonstrated the detection of single virion avian influenza viruses at a concentration of 10^−17^ M, using a chip with silicon nanowire sensors. Shen F. et al. [9], employed a sensor chip, based on silicon nanoribbons, fabricated by chemical vapor deposition (CVD). With such a chip, these authors demonstrated the detection of influenza A viral particles at a concentration of 29 viruses/μL (VP/µL) in 100-fold diluted clinical exhaled breath condensate (EBC) samples [9]. Once again, the authors of the above-mentioned papers used sensor chips of p-type conductance for the detection of target viral particles. Herein, we demonstrated the use of sensor chips of n-type conductance for the detection of influenza virus particles. It should be emphasized that, in contrast to the above-cited works, our sensor chips are fabricated using a complementary metal-oxide-semiconductor (CMOS)-compatible technology, thus allowing simpler transition to their mass production. In our experiments, the lowest detectable concentration of the target viral particles amounted to 6 × 10^−16^ M (10^4^ VP/mL).

## 2. Materials and Methods

### 2.1. Equipment

Nanoribbon detector (Russia), UV Ozone Cleaner—ProCleaner™ Plus (Ossila Ltd., Sheffield, UK), Piezorray micro-arraying system (PerkinElmer, Inc., Waltham, MA, USA), 10-channel data collection and storage system (Agama + JSC, Moscow, Russia).

### 2.2. Chemicals

The cross-linking agent 3,3′-dithiobis (sulfosuccinimidyl propionate) (DTSSP) was purchased from Pierce (Waltham, MA, USA). The following chemicals were also used: Potassium phosphate monobasic (KH_2_PO_4_), hydrofluoric (HF) acid, 96% ethanol (C_2_H_5_OH) (Reakhim, Moscow, Russia); 3-aminopropyltriethoxysilane (APTES) (Sigma Aldrich, St.-Louis, MO, USA); isopropanol 99.9% (C_3_H_8_O) (Acros Organics B.V.B.A., Geel, Belgium). Deionized water was obtained with a Simplicity UV purification system (Millipore, Molsheim, France).

### 2.3. Proteins and Viral Particles

Murine monoclonal antibodies against hepatitis B virus antigen (HBsAg) (clone NF5), affinity purified to 96%, were obtained from Federal State Budget Institution State Research Center Immunology Institute of the Russian Federal Biomedical Agency (Moscow).

Antibodies against the influenza A virus (subtype A (H1N1) pdm09) were isolated from hyperimmune ferret serum. The antibodies specimen were isolated from animal hyperimmune serum, and non-specific hemagglutination inhibitors were taken away. Influenza A virus (subtype A (H1N1) pdm09) was cultivated in chicken embryo allantoic cavity, concentrated and purified by ultracentrifugation, and deactivated with β-propiolactone (Merck).

### 2.4. Nanoribbon Detector

**Figure 1:**
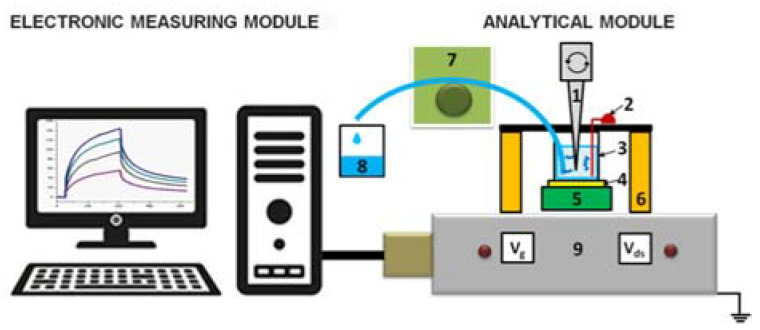
Schematic of the nanoribbon detector. Numbers indicate the main elements of the detector: 1—stirrer, 2—Pt electrode, 3—measuring cell, 4—silicon-on-insulator nanoribbon (SOI-NR) sensor chip, 5—chip holder, 6—measuring cell holder, 7—peristaltic pump, 8—waste container, 9—ten-channel data collection and storage system.

NR detector is a system consisting of two main modules: analytical and electronic measurements modules (Figure 1).

**Figure 2:**
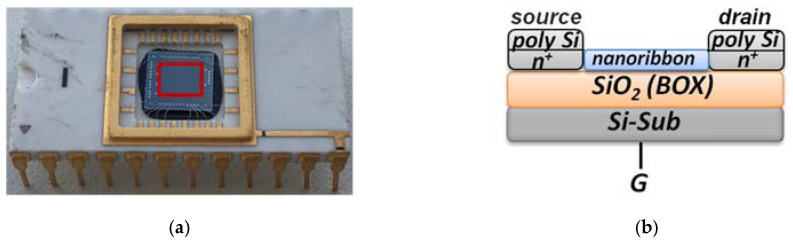

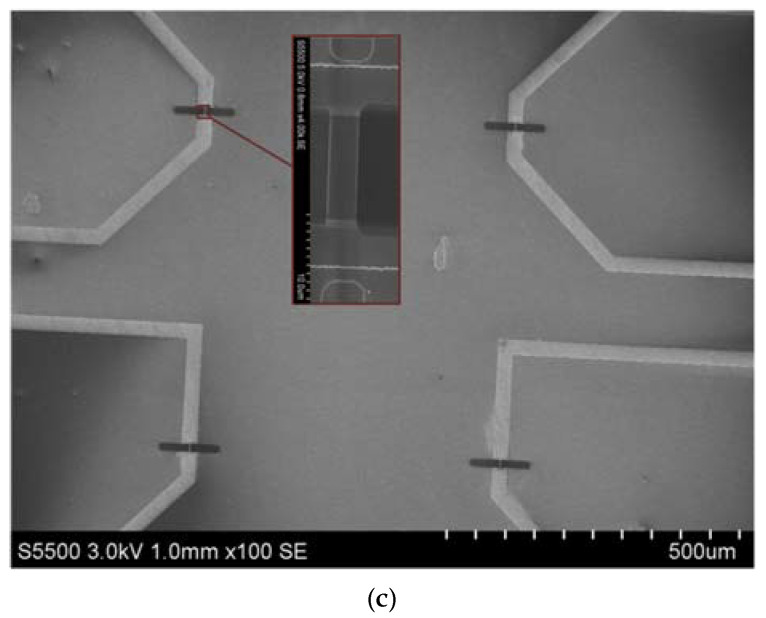
Photographic images of an assembled SOI-NR chip (top view), red square indicates the sensitive area (**a**); schematic representation of a cross-section of an individual SOI-NR with highly doped polysilicon source-drain contacts (**b**); SEM image of a section of the sensitive area of the SOI-NR chip, inset displays the SEM image of an individual NR (**c**).

The analytical module consists of a 500-µL measuring cell, whose bottom is a «silicon-on-insulator» (SOI) nanoribbon chip integrated in the standard micro scheme; 10 n-type NRs are organized in pairs on the surface of the chip. NRs’ characteristics were as follows: the cut-off Si layer was 32-nm-thick, buried oxide (BOX) was 300-nm-thick; NRs’ width (w) was 3 µm, their thickness (t) was 32 nm, and their length (l) was 10 µm (Figure 2) [10,11,12].

SOI nanoribbon chips #1 and #2 were used in our work. The diameter of the sensitive area was ~2 mm. The solution was mixed in the measuring cell with an agitator at a speed of 3000 rpm.

The module of electronic measurements is designed to simultaneously detect signal from 10 NRs placed on the chip, and to visualize the signal on the computer in the form of sensograms during the assay in real-time mode. Transformation of the detected signal into a digital one, as well as the analysis and visualization of measurement results (their presentation in a graphic form) were carried out using a specialized software (Agama + LLC, Moscow, Russia).

### 2.5. Chemical Modification of SOI Nanoribbon Chip Surface

Chemical modification of the NR surface included the preliminary purification and the silanization procedure using 3-aminopropyltriethoxysilane (APTES). At the preliminary purification stage, mechanical impurities were cleaned from the surface of SOI nanoribbon chips with isopropanol. Then, the surface of SOI nanoribbon chips was treated in a solution containing HF acid and ethanol in the proportion of 1:50 to remove natural oxidation formed during open storage. In order to form hydroxyl groups on the NR surface, the chip was put in an ozone generator. The silanization process was conducted in APTES vapor for 20 h at room temperature. On the next step, the surfaces of SOI nanoribbon chips were washed with ethanol [13].

### 2.6. Sensitization of SOI Nanoribbon Chips

**Figure 3:**
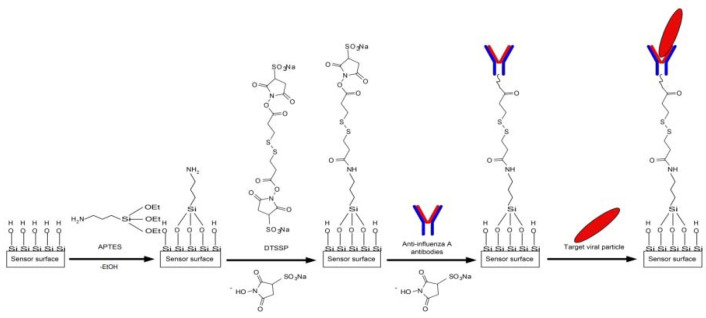
Schematic of the chemical modification of the sensor surface with APTES and its further sensitization with antibodies against the influenza A virus.

To provide the specificity of influenza A virus detection, we sensitized the NR sensor surface by covalent immobilization of antibodies against influenza A virus (working NRs) and hepatitis B virus (control NRs). Both types of antibodies were immobilized following the same protocol. The sensor surface was activated with DTSSP cross-linker solution, and then the antibodies were immobilized onto the activated surface of individual NRs as described elsewhere [14] (Figure 3).

To account for a non-specific adsorption of target particles, NRs with immobilized antibodies against the influenza A virus were chosen on each chip; they were used as working sensors. In contrast, control NRs (located on the same chip) were sensitized with the antibodies against HBsAg.

Specific antibodies bind to a particular antigen epitope. The energy of such a binding is high, exceeding dozens of kcal/mol [15]. This is why specific antibody–antigen complexes are quite stable: the equilibrium constant of formation of such complexes typically makes up 10^6^ to 10^12^ M^−1^ [16,17]. Non-specific binding of antibodies is possible, but it results in the formation of weak van der Waals bonds with energy lower than 1 kcal/mol [15,18]. These interactions are much weaker than chemical bonding, and such complexes are unstable, dissociating very quickly.

### 2.7. Preparation of Target Antigen Solutions

Antigen (influenza A virus) solutions with concentrations from 10^3^ to 10^10^ VP/mL were prepared immediately before the biosensor measurements by subsequent tenfold dilution with the processing buffer solution (1 mM potassium phosphate buffer, pH 7.4). On each dilution step, the solutions were incubated in a shaker for 30 min at 10 °C and 600 rpm.

### 2.8. Electrical Measurements

**Figure 4:**
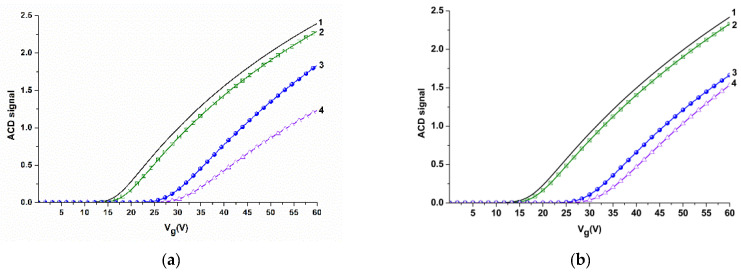
Volt-ampere characteristics obtained for NR sensors before and after their sensitization with molecular probes in air, in water, and in potassium phosphate buffer for NR#6 (with immobilized antibodies against influenza A virus) (**a**), and for NR#2 (with immobilized antibodies against HBsAg) (**b**). Experimental conditions: 1 mM potassium phosphate buffer, pH 7.4, *V_g_* 0 ÷ 60 V, *V_ds_* 0.1 V, liquid volume in the cell 300 μL. Black line (1) indicates volt-ampere characteristics (VAC) curves obtained for silanized NRs in air; green line (2) indicates VAC curves obtained for NR sensors with immobilized antibodies in air; blue line (3) indicates VAC curves obtained for NR sensors with immobilized antibodies in water; magenta line (4) indicates VAC curves obtained for NR sensors with immobilized antibodies in potassium phosphate buffer.

Electrical measurements were performed using a 10-channel data collection and storage system (Agama + JSC, Moscow, Russia), as described elsewhere [19]. The time dependencies of the drain-source current *I_ds_*(*t*) (i.e., the dependencies of the drain-source current *I_ds_* on time *t* at constant gate voltage *V_g_*) were measured at *V_g_* = 55 V and *V_ds_* = 0.1 V. The operating point *V_g_* value was selected based on volt-ampere characteristics measured in the buffer (Figure 4). The *V_ds_* was selected following the recommendations of the data collection and storage system and the SOI nanoribbon chip manufacturers.

To increase the time stability of the NR detector operation, we used an additional Pt electrode, immersed into the solution in the measuring cell, similar to [10,12,20]. Moreover, it was demonstrated that the injection of aqueous solutions into the measuring cell can induce an electric charge, which can influence the character of initial parts of binding curves before the so-generated charge drains to the ground. This is why the use of the Pt electrode levels a charge, which may be induced in the solution injected into the measuring cell [21].

### 2.9. Measurements with the Use of a NR-Detector

SOI nanoribbon chip #1 was used in experiments on the detection of the target viral particles at the following concentrations: 10^3^ VP/mL; 10^4^ VP/mL; 10^5^ VP/mL; 10^7^ VP/mL. SOI nanoribbon chip #2 was used to detect the target particles at concentrations of 10^9^ VP/mL and 10^10^ VP/mL. The analyzed solution (7 µL in 1 mM potassium phosphate buffer), containing the target particles, was added into the measuring cell of the NR detector, containing 100 µL of 1 mM potassium phosphate buffer. Control experiments were performed under similar conditions, but using pure, VP-free buffer solution instead of VP solution.

Registration of the NR detector signal was performed in real-time mode. The data collected were presented in the form of sensograms: time dependencies of a non-dimensional value of the current were expressed in relative units. The signal in relative units was calculated for each NR as the Δ*I_ds_*/|*I_ds_*_0_| relation of the intensity of current (*I_ds_*) in a given period of time and the intensity of current (*I_ds_*_0_) at the initial point. The signal obtained in control experiments was deducted from the absolute signal, which was detected during the analysis of the solution containing influenza A virus particles. Then, the differential signal from the working NR sensor (with immobilized antibodies against influenza virus), and the control sensor (with immobilized antibodies against the hepatitis B virus surface antigen), was calculated. The detection of the target viral particles was carried out in a low-salt buffer (1 mM potassium phosphate buffer) in order to avoid Debye screening effect [22,23].

## 3. Results

### 3.1. Sensitization of the NR Sensor Surface

Sensitization of the NR sensor surface was performed in order to provide biospecificity of the target particles detection. The sensitization was carried out by covalent immobilization of molecular probes—antibodies against the target particles (see Sensitization of SOI nanoribbon chips in Materials and Methods). The efficiency of this procedure was determined by comparative analysis of volt-ampere characteristics (VAC) before and after the molecular probes’ immobilization. Figure 4 displays the typical VAC curves obtained for NR#6 (with immobilized antibodies against influenza A virus), and NR#2 (with immobilized antibodies against HBsAg).

From the curves presented in Figure 4, one can see that, in air, an insignificant (~ 2 V) shift in the VAC to the right is observed for antibody-sensitized NRs—in comparison to silanized NRs without immobilized antibodies. This indicates the successful immobilization of molecular probes onto the NR surface.

In aqueous medium (water or potassium phosphate buffer), this VAC shift makes up ~10 to 12 V for NR sensors with immobilized antibodies (Figure 4a,b). The significant shift in VAC in water and in potassium phosphate buffer is probably induced by additional negative charge, provided by the presence of OH-groups.

Since subsequent experiments (registration of *I_ds_*(*t*) dependencies) were performed in real time in 1 mM potassium phosphate buffer, the VAC data obtained in the buffer were used for the selection of operating point *V_g_* for these experiments. As one can see from Figure 4, *V_g_* = 55 V is optimal in our conditions, as the risk of displacement of the signal to the «closed transistor» area (where the *I_ds_* value makes up ~10^−10^A) is decreased.

### 3.2. Biospecific Detection of Influenza a Virus with the NR Detector

**Figure 5:**
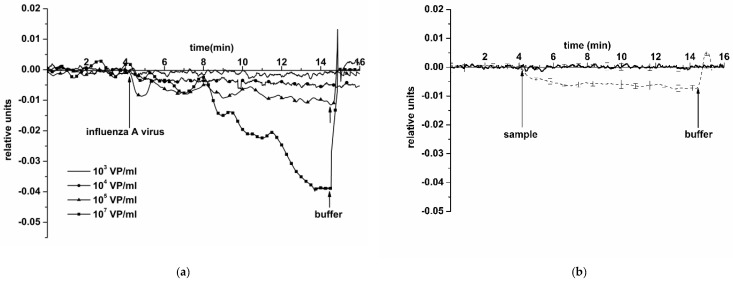
Typical sensogram curves obtained upon the detection of influenza A virus in buffer using the NR sensor chip of n-type conductance with covalently immobilized antibodies: (**a**) sensograms obtained at various concentrations of target viral particles; concentration of viral particles in the solution is 10^3^ VP/mL (solid line without markers), 10^4^ VP/mL (circles), 10^5^ VP/mL (triangles), and 10^7^ VP/mL (squares); (**b**) sensograms obtained upon analysis of the solution with target particles concentration of 10^4^ VP/mL (thin dashed line), and upon analysis of (viral particle)-free buffer (thick solid line); number of technical replicates *n* = 3. Experimental conditions: 1 mM potassium phosphate buffer, pH 7.4, *V_g_* 55 V, *V_ds_* 0.1 V, solution volume in the cell 107 μL. NR is sensitized with antibodies against influenza A virus. Arrows indicate addition of the sample solution and washing with a pure potassium phosphate buffer.

Figure 5 displays typical sensograms obtained in the course of the detection of influenza A virus at a concentration of ~10^3^, 10^4^, 10^5^, and 10^7^ VP/mL using an SOI NR sensor chip #1 of n-type conductance. The curves in the figure indicate that addition of the solution, containing influenza A virus particles, into the biosensor measuring cell induces a decrease in the electric current through the NR-sensor, sensitized with antibodies against the target particles. This decrease is obviously caused by the adsorption of negatively charged molecules onto the NR surface. Moreover, it is worth mentioning that the intensity of the current through the NR sensor decreases when increasing the concentration of the target viral particles.

Control experiments were conducted using a pure protein-free potassium phosphate buffer (pH 7.4) instead of an influenza A virus solution (thick solid line in Figure 5b). The response from the sensor was virtually indistinguishable. This confirms that, at neutral pH 7.4, the response signal observed in experiments with an influenza A virus solution is indeed caused by a biospecific interaction between the sensor-immobilized antibodies and the target molecules.

**Figure 6:**
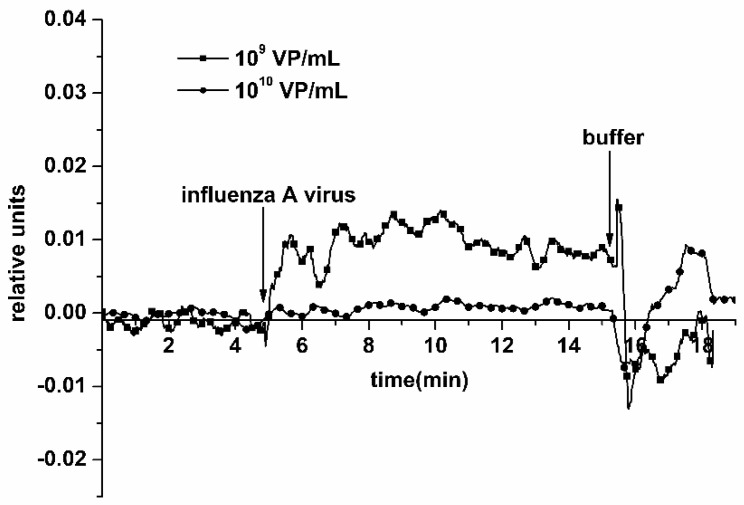
Typical sensograms obtained upon the detection of influenza A virus with the use of the NR detector. Experimental conditions: 1 mM potassium phosphate buffer, pH 7.4, *V_g_* 55 V, *V_ds_* 0.1 V, solution volume in the cell 107 μL; NR is sensitized with antibodies against influenza virus. Concentration of viral particles in the analyzed solution is 10^9^ VP/mL (squares) and 10^10^ VP/mL (circles). Arrows indicate addition of the sample solution and washing with pure potassium phosphate buffer.

The addition of the solution with a virus concentration of 10^9^ and 10^10^ VP/mL into the measuring cell led to a stabilization of the current at its permanent, maximum possible levels, which corresponds to the transistor saturation mode and overabundance of viral particles on the NR surface. Typical sensograms, obtained upon the detection of influenza A virus at 10^9^ VP/mL and 10^10^ VP/mL concentrations using an antibody-sensitized NR sensor chip, are shown in Figure 6. From this figure, one can see that the addition of a 10^9^ VP/mL VP solution into the cell leads to an increase in the biosensor signal. The increase in the NRs’ conductance—rather than its decrease, observed earlier at lower viral particle concentrations—probably occurs due to the NR surface charge exchange, which can be caused by the formation of aggregates of viral particles and their cumulative positive electric charge.

Washing the chip surface between measurements allowed efficient regeneration of the NR sensor surface, when continuously analyzing solutions at concentrations of the target viral particles within the range between 10^3^ and 10^7^ VP/mL.

**Figure 7:**
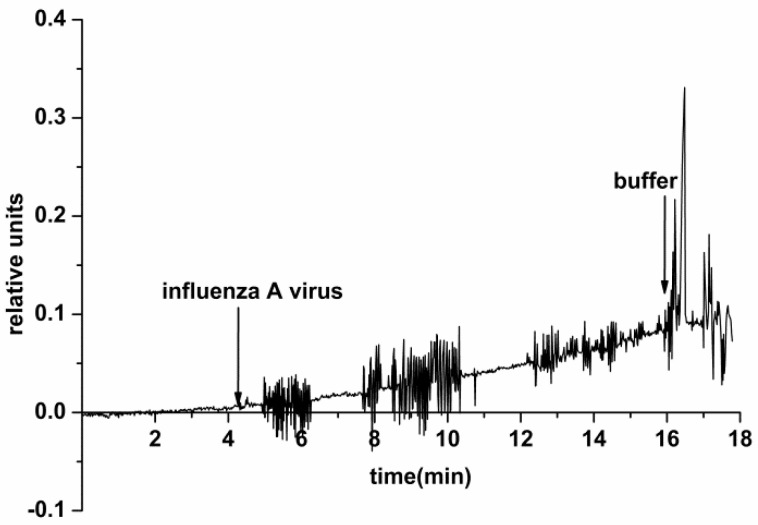
Typical sensogram obtained upon the detection of influenza A virus with the use of the NR detector. Experimental conditions: 1 mM potassium phosphate buffer, pH 7.4, *V_g_* 55 V, *V_ds_* 0.1 V, solution volume in the cell 107 μL. NR is immobilized by antibodies against influenza A virus. The concentration of the target viral particles in the solution is 10^3^ VP/mL. Arrows indicate addition of the sample solution and washing with pure potassium phosphate buffer.

Our experiments with the use of an SOI nanoribbon chip #2 have, however, indicated an insufficiently efficient surface regeneration by washing after working with a higher concentration of viral particles (10^10^ VP/mL). In the course of subsequent analysis of a solution with a lower concentration of the target particles (10^3^ VP/mL), the biosensor signal increased and, in single assays, signal fluctuation was observed (Figure 7).

The electric current fluctuations can appear due to the process of adsorption/desorption of single VPs onto/from the NR surface. In the case of experiments with control VP-free solutions (conducted before the experiments with VPs), only a noise signal was registered. This indicates a specific interaction between the NR-immobilized antibodies and the target viral particles from the analyzed solution.

## 4. Discussion

For the fabrication of NR sensors, two opposite approaches are distinguished: «bottom-up», and «top-down». In methods utilizing the «bottom-up» approach, the catalytic or non-catalytic NR growth by gas-phase epitaxy [24,25,26] or molecular-beam epitaxy [27] are employed most often. In these methods, the use of a metal particle (typically, gold) as a catalyst of the NR growth is required. Such a process is quite laborious. The proper formation of electric contacts with the sensor structures is the most complex task. Moreover, achieving high yields of high-quality sensor chips represents a problem.

The sensor chips, used in our experiments, were fabricated using a «top-down» approach. In this approach, the process of sensor fabrication represents a transition from a large-volume material to nanometer-size structures, which is achieved by nanostructuring of this material. Such an approach to the fabrication of «silicon-on-insulator» (SOI) structures is the most promising owing to the following advantages: (1) relative ease of manufacture (lateral structuring of silicon nanolayers is only required); (2) use of the substrate of the SOI structures as a control gate (this allows for controlling the sensitivity); (3) compatibility with the standard complementary metal-oxide-semiconductor (CMOS) technology. The latter allows one to combine the formation of sensor elements with control and information processing circuits on one crystal. On the one hand, this will allow for the fabrication of electronic detectors with high sensitivity and speed of operation; and on the other hand, this will allow for the development of portable devices (affordable for individual users) for rapid monitoring of the level of health and early revelation of diseases in humans.

The NR detector represents an advanced device for highly sensitive detection of viruses. The NR detector is able to detect even single adsorbed viral particles per sensor element [7]. Another advantage of the NR detector is its capability to directly detect the target particles. This eliminates the use of additional labels and, accordingly, simplifies the analysis, providing high sensitivity. Unlike PCR, the use of the NR detector does not require amplification and, accordingly, is much less sensitive to sample contamination, and avoids false-positive results [28,29].

In earlier papers, NR detectors for the detection of viruses were described [7,8,9]. In our present study, we employed a n-type chip, produced according to the CMOS-compatible technology, which is cheaper than the CVD technology. Our paper examines the possibility of detecting influenza virus particles in a mixture of different viral particles often used in diagnostic sets. We have demonstrated that the virus particles can be detected in a buffer solution using a highly sensitive NR detector (at ~10^−16^ M) in real-time mode without using special labels. It is worth noting that repeated use of the NR sensor chip while working with high concentrations of viral particles requires attention when transferring to measurements at low concentrations. Thus, signal fluctuation was observed upon the analysis of a low-concentration solution of viral particles (10^3^ VP/mL) after working with high concentrations (10^9^ to 10^10^ VP/mL). We suppose that an incomplete regeneration of the sensor surface—and, accordingly, the presence of residual viral particles on the surface—leads to signal fluctuations (Figure 7). Another cause of such fluctuations can be the fact that, at such a dilution, single viral particles are stochastically adsorbed onto the NR surface. Detection in a ~10^3^ VP/mL solution can make conditions, in which single viral particles can stochastically adsorb onto the positively charged NRs.

Thus, the use of a SOI NR chip for detection of influenza virus at lower concentrations is not acceptable after the chip was subjected to higher VP concentrations. In modern medical and virological common laboratory practice, expendables are only meant to be used once. This is explained by the possibility of pathogen particles remaining on the expendables even after a thorough washing, causing an uncontrolled emergence of a pathogen, leading to serious consequences.

## 5. Conclusions

In our experiments, we have employed sensors based on SOI structures, fabricated by lateral nanostructuring. The advantage of these sensors is in the use of buried oxide of the SOI structures as gate dielectric, and the silicon substrate as a backside gate. This allows the NRs to function as silicon nanotransistors with two gates (liquid electrolyte and conductive substrate). Accordingly, upon adsorption of target analyte particles onto the sensor, the modulation in the NR conductance is registered within a wide range of analyte concentrations.

Our present study is aimed at the detection of influenza A virus using sensor chips based on silicon-on-insulator nanoribbon structures. The results obtained herein have indicated that our NR detector allows for the real-time detection of influenza A viral particles. NR sensor chips of n-type conductance have been fabricated using CMOS-compatible technology, with the use of gas-phase etching and lithography.

Our NR detector has been demonstrated to be capable of detecting influenza A virus particles with high sensitivity at the level of 10^4^ VP/mL, which corresponds to 6 × 10^−16^ M, in a mixture used for diagnostic tests. The results obtained herein indicate that NR detector represents a prototype of a diagnostic device, which offers good opportunities for implementation in medical practice.

The use of high-quality monocrystalline silicon instead of polysilicon as a material for NR sensor fabrication provides stable electric characteristics of the NR sensors in solution and, therefore, high sensitivity. This allows one to detect target particles at ultra-low (subfemtomolar) concentrations, which is particularly important in both fundamental and applied research in proteomics and metabolomics for the determination of the content of specific proteins and metabolites in the human body.

## Data Availability

The datasets generated during and/or analyzed during the current study are available from Y.D.I. on reasonable request.
